# Point-Of-Care Ultra-Sound (POCUS) for suspected surgical abdominal conditions in rural India–a mixed-method health needs assessment

**DOI:** 10.1371/journal.pgph.0001233

**Published:** 2023-01-06

**Authors:** Owen Thomas, Noel Aruparayil, Jesudian Gnanaraj, George Mathew, Kavikumar Nagarajan, Beth Shinkins, David Jayne

**Affiliations:** 1 Leeds Institute of Medical Research at St. James’s, University of Leeds, Leeds, United Kingdom; 2 Association of Rural Surgeons of India, Chepauk, India; 3 Shanti Bhavan Medical Center, Jharkhand, India; 4 Aarohi, Uttarakhand, India; 5 Academic Unit of Health Economics, University of Leeds, Leeds, United Kingdom; Harvard Medical School, UNITED STATES

## Abstract

**Introduction:**

Evidence has shown a trend towards rising mortality from surgical abdominal conditions with increasing distance from hospitals in Eastern India. It has been claimed that imaging modalities such as portable ultrasound are ideal for use in rural areas of developing countries for diagnosis and management of a variety of acute abdominal conditions. This mixed-methods study aimed to explore the need for increased access to POCUS in rural, hard-to-reach populations within India, and to identify barriers that may exist to increasing this access.

**Methods:**

This mixed-methods study utilized a health needs assessment framework. Quantitative data was extracted from medical records within two outreach camp organisations supporting hard-to-reach populations in Uttarakhand and Jharkhand to provide contextual background statistics. Qualitative data was collected using semi-structured interviews taken from employees of each outreach camp. A framework analysis was employed to analyse the qualitative data. Both datasets were analysed in parallel.

**Results:**

Twelve semi-structured interviews were undertaken, and 3 months of medical records were reviewed. Themes that presented themselves from the data included the use of camps for case-finding of conditions amenable to routine surgery, a need for POCUS to help with this diagnostic process, a perceived high disease burden from renal calculi, and complex cultural and legislative barriers to POCUS specific to this setting.

**Discussion:**

POCUS was considered a useful tool in expanding access to surgery for these settings and finding ways of overcoming cultural and legislative barriers to its use should be of high priority. POCUS should be highly portable, robust, easily repairable, and battery operated. For telemedicine to be fully utilised, signal quality will have to be proven to be reliable.

## Introduction

There is a need to increase capacity for surgical care provision in low and middle-income countries. The Lancet Commission on Global Surgery reported that 143 million extra surgical conditions a year go untreated, causing untold suffering and leading to an economic loss estimated around US$ 12.3 trillion [1]. Acute abdominal and pelvic conditions have been highlighted as priority surgical conditions requiring expansion of provision. India is a lower middle-income country that has widely varying access to surgery. Dare et al. 2015 showed a steep climb in standardised mortality with increasing distance from well-resourced hospitals in India, highlighting significant problems with death rates of those with surgical abdomen conditions in resource-poor eastern Indian states [2].

Blood, urine, and imaging tests have been recognised as “vital” investigations for the acute abdomen [3]. Point-Of-Care Tests (POCT) could begin to fulfil this role in rural clinics where centralised laboratory infrastructure is less accessible. The ‘Lancet Commissions; Global surgery 2030’ team recognised the need for such innovations [1]. Despite this, there has been limited comprehensive research into their use in low-resource settings.

‘Point-Of-Care Ultra-Sound (POCUS) technology is an imaging modality with unique advantages over other imaging forms such as CT and MRI, including a lack of radiation, instant results, smaller set-up costs, and increasing portability [4,5]. It is now ubiquitous among most hospitals in high-income countries and is a crucial tool in the evaluation of traumatic abdominal conditions, with particular efficacy in the assessment of biliary colic, abdominal masses, and localised abdominal pain with deranged blood tests [6]. Maru et al., 2010 argue that imaging modalities such as portable ultrasound and radiography are ideal for use in rural areas of developing countries for diagnosis and management of a variety of acute abdominal conditions [7]. They concluded that when present in Primary Healthcare Centres (PHCs), they can meet the imaging needs of 90% of the population [7]. The WHO conclude that these imaging systems are vital in primary care and in situations where other imaging is impractical [8,9]. Despite these benefits, the spread of this technology to resource-limited settings has been hampered by location-specific requisites related to climate, energy supply, device size, image quality, and the need for on-site specialists to interpret the findings [10–13].

Many of these technological issues have been addressed over the last decade, and enhanced devices with additional functionality are starting to be utilised. Eptein et al., 2018 showed successful implementation of ‘pocket-sized’ ultrasound devices in rural hospitals in sub-Saharan Africa, which were operated by physicians with only basic POCUS training and supported by telemedicine support [14]. Wydo et al., 2015 provide a review of the literature of portable ultrasound use in disaster settings and concluded there is evidence suggesting ultrasound images can be reliably transmitted via commercial satellites and low-bandwidth internet connections for remote review [15].

Within India, Jha *et al*., 2006 have shown that pre-natal selective abortion of female foetuses was likely to account for a reduction in 0.5 million female births in 1998, leading to a significant national sex ratio reduction [16]. In part due to this, India’s access to ultrasound technology is now controlled by the “Pre-Conception and Pre-Natal Diagnostic Techniques Act (PNDT)”. This Act is intended to curb sex determination and therefore, potential female foeticide; however, others have commented on the unintended side-effect of limiting access to healthcare in rural populations for other medical conditions, whose local practitioners often lack the resources to obtain the licences required [17]. There is on-going debate about the efficacy these changes have made to the imbalances [18].

Given the established benefits of POCUS, the aim of this study was to explore if there is a need for increased access to POCUS in rural, hard-to-reach populations within India, and to identify barriers that may exist to increasing this access.

## Methods

This mixed-methods study used a health needs assessment framework–as described by Christianson et al., 2013 and the UK Health Development Agency–to examine the need for POCUS in suspected surgical abdominal presentations within hard-to-reach, rural, Indian outreach camps [19,20]. This involved creating a situational analysis of the current disease burden in this setting, exploring the expressed unmet health needs, and exploring if POCUS was a feasible technology to meet these needs. Two comparison camps were selected: a camp ran by a state-approved Non-Governmental Organisation (NGO) ‘Aarohi’ with access to POCUS on their Mobile Medical Unit (MMU), supplying a mountainous population in the northern Indian state of Uttarakhand; and a camp run by the NGO ‘Shanti Bhavan Medical Center’ (SBMC) without access to POCUS in the rural areas of Eastern Indian State of Jharkhand. At the time of the study, two ultrasound devices were available within Aarohi: a stationary device at the basecamp facility (SIEMENS–ACUSON X300PE, SN—351643, Model number– 10566144); and a mobile device carried on the MMU (SIEMENS—SONOLINE—G20 –Serial number: 09402828). The devices were licensed to be operated by five authorised radiologists, who were all volunteer staff who lived outside the region and would make outreach trips to operate the device during pre-planned clinics and mobile camps–no other staff were allowed to operate the technology. POCUS would be undertaken at the request of a clinician usually during the same day as the scan, based on clinical need, and would be interpreted simultaneously. This study received ethical approval from the ‘University of Leeds, UK’ (MREC 19–007) and the ‘Martin Luther Christian University’, India (WI/IUREC/EA/272/2015-5971). It complies with the ‘Consolidated criteria for Reporting Qualitative research’ Checklist [21].

### Quantitative

Data was collected from pre-existing medical records for January-May 2019 in Aarohi and September-November 2019 in SBMC. Non-surgical abdomen cases were excluded. Anonymised data was recorded on presenting symptoms, diagnosis, and use of POCT, along with collated national/regional records containing population demographics of the two regions [22–24]. Simple descriptive statistics were used to describe the demographics of the population using the two camps, along with the frequency of symptom presentation, recorded diagnosis, and medical tests utilised.

### Qualitative

Qualitative data was collected by the primary researcher (OT) in the form of semi-structured interviews taken from a purposeful sample of team members involved in the rural outreach camps, along with field notes from the researcher. The interviewing researcher was a medical doctor with prior experience in semi-structured interviews. The researcher spent a week with both teams to establish rapport and improve the researcher’s understanding of the facilities. An interview guide piloted within the research team and a lead at each site was used. Participants were approached face-to-face, given verbal information about the project, and given time to review written information. Written informed consent was gained, and interviews took place alone at the clinic. Repeat interviews were not utilised, and whilst transcripts and conclusions were not returned to participants, areas of clarification were sort with participants by the researcher. Audio recordings were employed, with interviews lasting 20–40 minutes.

**Fig 1:**
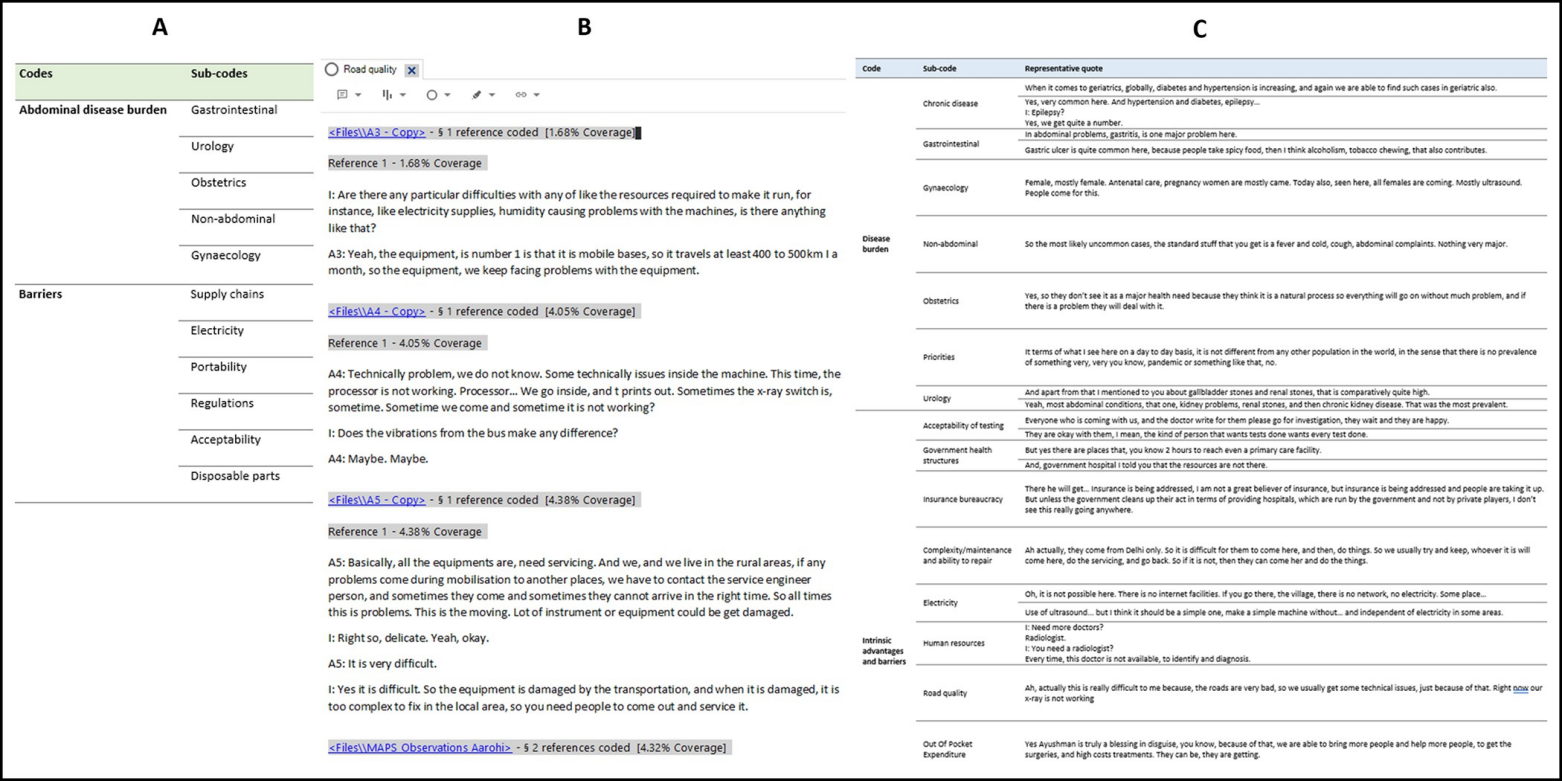
An illustration of sections of the a-priori codex, the coding process in N-vivo, and sections of the charting process. A–An example of the a-priori coding schedule; B–An example of the coding process in Nvivo–version 11; C–An example of the final coding schedule with quote examples.

A framework analysis was chosen to analyse the data, following the steps defined by Ritchie and Spencer, focusing on a content analysis methodology [25,26]. These stages included transcription, familiarisation with the dataset, creation of a thematic framework, indexing, charting, and mapping. A codex was produced from *‘a priori’* themes produced from a background narrative literature review conducted by primary researcher, and expanded on through inductive themes arising from the data [27]. A second reviewer (NA) assessed a randomly selected set of interviews, and disagreements around coding were settled by discussion. N-vivo (Version 12) and Excel (version 2201) were used to assist this process [28]. Fig 1 illustrates examples of the a-priori codex, the coding process in N-vivo, and sections of the charting process.

## Results

Twelve semi-structured interviews were undertaken, equally split between both sites. The participants consisted of two administrators, seven doctors, and three other healthcare professionals. Results are presented through themes and sub-themes.

### Quantitative data presentation and perceived disease burden

In both locations, most participants felt that there were no significant differences in local disease burden compared to other areas. A few participants in both camps did highlight a high prevalence of chronic abdominal pain and most participants identified a markedly high incidence of kidney stones and gallstones. One participant in both locations felt the high frequency of these diseases was induced by local people having a poor thirst reflex.

Aarohi1: *“So we have a little bit of a problem here in terms of thirst mechanisms and drinking water*. *So most local people do not drink too much of water*. *So that is a little more prevalent of what we call stones inside the body*.*”*

In SBMC many participants highlighted that there was a significant burden of disease from gynaecological and obstetric conditions, with most participants suggesting that the lack of ultrasound in the region made diagnosing these diseases difficult and imposed a significant limitation on Antenatal Care (ANC) within the region. It was felt by a few participants that this issue was particularly acute in tribal populations.

SBMC5: *“Oh yes*, *antenatal care is a very important part of*, *so our fertility rates are high*, *pregnancy rates are high*, *so yeah*, *but not everyone would seek hospital or healthcare because there are traditional birth attenders*, *and people that look after antenatal and deliver*, *birth*. *Gynaecological problems*, *yes*, *that is a significantly large proportion of the surgical burden*.*”*

Data collated on the demographics of the population in the two rural states can be seen in Table 1. Data from Aarohi was reviewed from January-May 2019 for each MMU trip through the region covering a population of 54,857 within 125 villages, excluding March 2019 due to clinic cancellation. During this time, 70 patients were seen with an abdominal presenting complaint (75.7% female). The most common presenting complaint was abdominal pain (68.5%), with abnormal ANC check-ups representing 20% and the remaining patients presenting with irregular bleeding, loose stools, and loss of appetite (Table 2). In 80.0% of abdominal presentations, some form of POCT was used. In 98.2% of these occasions, the POCT utilised was POCUS (Table 3).

**Table 1:** Demographics and key indicators from Uttarakhand and Jharkhand ((Ministry of Health & Family Welfare, 2018; World Bank Group, 2016; Work Bank Group 2017).

Descriptor	Uttarakhand	Jharkhand
**Demographics**		
**Geographic Area**	53,484 sq. kms	79,714 sq. Kms
**Number of villages**	16,793	32,394
**Total Population**	10,116,752	32,988,134
**Rural population**	69%	76%
**Child Sex Ratio (per 1000 male)**	894 (948 in 1991)	948
**Density- per sq. km.**	189	414
**Education and employment**		
**Literacy Rate (6+ Popn.)**	79.63%	66%
**Agricultural employment**	49%	50%
**Adults with secondary school education**	38%	28%
**Jobs and poverty**		
**Population below the poverty line**	11%	37%
**Real GSDP per capita**	59327 rupees	27779 rupees
**Power**	95%	69%
**Drinking water**	61%	29%
**Social group structure within the state**		
**Percentage of Scheduled Tribe**	4%	27%
**Percentage of Scheduled caste**	20%	14%
**Percentage of other backward caste**	17%	44%
**Percentage of general social group**	59%	15%

**Table 2:** Data from SBMC* and Aarohi** on abdominal presentations.

SBMC (n = 49)		Aarohi (n = 70)	
**Presenting complaint**	**Total**	**Presenting complaint**	**Total**
Abdominal pain	28 (57%)	Abdominal pain	49 (70%)
Urinary symptoms	15 (30%)	Antenatal care check-up	14 (20%)
Abdominal/pelvic mass	8 (16%)	Blank	3 (4%)
Back pain	4 (8%)	Irregular bleeding	2 (3%)
Scrotal symptoms	4 (8%)	Loose stools	1 (1%)
Distention	4 (8%)	Loss of appetite	1 (1%)
Haematuria	4 (8%)		
Shortness of breath	2 (4%)		
Vomiting	2 (4%)		
Rectal pain	1 (2%)		
Blood in stool	1 (2%)		
Jaundice	1 (2%)		
Loss of appetite	1 (2%)		
Vaginal discharge	1 (2%)		

**Table 3:** Data from SBMC* and Aarohi** on types of POCT used during consultation.

SBMC		Aarohi (n = 70)	
Type of POCT used	Total	Type of POCT used	Total
N/A	N/A	Ultrasound	55 (79%)
		None	14 (20%)
		Blood test	4 (6%)
		Hb test	3 (4%)
		Foetal doppler	2 (3%)
		Pregnancy test	1 (1%)

*SBMC data collected from Jan 19 to May 19.

**Aarohi data collected from Sept 19 to Nov 19.

Data from SBMC was reviewed from Sept 19 to Nov 19 inclusive, for all patients documented as seen by the outreach team. During this time, 49 patients presented with an abdominal complaint (36.7% female). The most common presenting symptom was abdominal pain (57.1%), followed by urinary symptoms (30%). Aarohi had 13 diagnoses of renal calculi and 5 cases of cholelithiasis, whilst SBMC had 9 renal colic diagnoses and no confirmed diagnosis of cholelithiasis (Table 4). The results allow us to approximate that within the Aarohi MMU catchment region, the incident of presenting to the MMU and getting a diagnosis of renal calculi was approximately 9.5 per 10,000 people per year.

**Table 4:** Data from SBMC* and Aarohi** on diagnosis recorded.

SBMC (n = 49)		Aarohi (n = 70)	
Diagnosis	Total	Diagnosis	Total
Renal colic	9 (18%)	Renal calculi	13 (19%)
Benign prostatic hypertrophy	4 (8%)	Gastritis	8 (11%)
Chronic Kidney Disease	4 (8%)	Breech presentation	6 (9%)
Gastritis	3 (6%)	cholelithiasis	5 (7%)
Haemorrhoids	3 (6%)	Appendicitis	4 (6%)
Hydrocele	3 (6%)	Worm infection	4 (6%)
Rectal Prolapse	3 (6%)	Anaemia	3 (4%)
Acid reflux	2 (4%)	Enlarged empty uterus	2 (3%)
Cystitis	2 (4%)	Uterine prolapse	2 (3%)
Inguinal Hernia	2 (4%)	Other	23 (33%)
Intestinal worms	2 (4%)		
Other	12 (24%)		

*SBMC data collected from Jan 19 to May 19.

**Aarohi data collected from Sept 19 to Nov 19.

### Background context on the workings of the two NGO mobile camps

In both locations, most participants highlighted three shared areas of focus for their workload:

**1) Case finding for non-emergency mobile surgeries**–Many interviewees highlighted that they were uniquely placed to screen the population for candidates with non-emergency surgical needs, which could be undertaken by teams of mobile surgeons at local centres at planned regular intervals depending on caseload.

Aarohi1: *“We also use the mobile medical unit to again dip into the population there*, *especially for people that need any surgical help*.*”*

**2) Health education**–participants in both locations stated that health education was a significant part of their workload. Aarohi focused on reproductive, maternal, new-born, child/adolescent health, along with educating village health workers to undertake health surveillance. SBMC focused on lifestyle and communicable diseases, whilst training lay community leaders to identify medical conditions.

SBMC1: *“Health education access*, *access to health education is poor*. *I think it is only the shanti bhavan community only that does some health education*.*”*

**3) Other medical issues (including emergencies)**–Both locations provided diagnostic and referral systems for routine and emergency medical issues.

In Aarohi, most participants felt that ANC was a large part of their workload, with one participant estimating that they were doing around 40 ANC POCUS scans per trip. All participants stated that the MMU provided the only ultrasound available within the region. Most contributors highlighted POCUS as being vital for routine ANC scans to monitor growth and identify abnormalities, investigate abdominal pain, and characterise lumps.

Aarohi2: *“And most of them are pregnant ladies*. *If*, *when you go with the team now*, *you will see that patients are coming from like so far”*.

In SBMC participants felt that the lack of ultrasound hindered their ability to provide ANC.

SBMC4: *“*: *Yeah*, *I mean there it* (POCUS) *would help see*, *the antenatal people would not have to travel so far… So probably there*, *that’s* (antenatal care) *one place I feel it may be definitely beneficial…”*

### Advantages and barriers for POCUS in the context of location-specific factors relating to the rural Indian setting

#### Health seeking behaviour

In Aarohi, half the participants stated that the attendees of the MMU believed in their doctors and were enthusiastic to have investigations done. POCUS was thought to be particularly desired; however, some participants felt that patient understanding of ultrasound may be limited and that often patients believed that POCUS was either therapeutic or was omniscient of their problems. One participant was concerned that the over-availability of POCT could lead to over-testing.

Aarohi2: *“They don’t know why the ultrasound is*, *sometimes that physician is prescribing*, *and they will say we do not need medicine*, *we need ultrasound*. *I don’t know what their impression of all the ultrasound is*, *they rely on to it*. *They are like please do ultrasound… Actually*, *our main power is the ultrasound machine…*. *Like today we do not have ultrasound*, *so we do not have patients*.”

Despite this, two participants in Aarohi felt that health seeking behaviour in the region was reduced in comparison to urban populations despite similar levels of education and literacy; it was felt that this was linked to an increase in fatalistic beliefs within the rural community, with people being more accepting of poor health and disease.

Aarohi3: *“And economically yeah*, *it is not that much backward*, *but when it comes to the health seeking behaviour*, *you can see wide differences*.”

Within SBMC, a key sub-theme throughout the discussions focused on the high population density of “tribals”, a term used to identify the ‘Scheduled Tribe’ communities forming roughly 26% of Jharkhand’s population (Table 1). Most participants highlighted tribals as being particularly vulnerable in terms of health seeking behaviour.

SBMC3: “*But here*, *in this tribal area… health seeking behaviour is little bit less here also*. *They think for everything of course*, *this is our fate*, *we cannot do anything because of our finance state*, *we cannot afford treatment…*

Several sub-themes arose as key barriers to tribals, and the rural population more broadly, seeking healthcare in Jharkhand. Most participants thought tribals were more likely to have fatalistic healthcare views, and several had concerns about tribal health beliefs around pregnancy. A few participants felt that a history of tribal exploitation within the region had caused this community to be suspicious of outsiders; however, one participant felt this was more due to a lack of education than suspicion. These participants also highlighted that trust was a very important commodity when trying to engage this community.

SBMC4: *“I*: *‘A few people have talked about their almost being a fatalistic way of looking at things*. *Is that…*?*’ S4*: *‘Yes to some extent*. *Life is a little cheap I think*, *that is the truth*.*’”*

SBMC1: *“So they have been exploited*, *they have been oppressed*, *they are far away*, *they are used to being sick*. *It is typical*.”

Most participants felt that poverty was a significant barrier to rural communities accessing healthcare, with poverty identified as widespread amongst the tribal population. They also highlighted barriers of non-direct costs associated with accessing healthcare, such as loss of earnings and travel costs.

SBMC4: *“Lack of money*, *that is the linchpin*. *The material world and money*, *lack of awareness*.”

SBMC1: *“Yes*, *it is difficult*. *In the farming*, *what should I say*, *in the farming period*, *people can’t let go of their wages*, *so if one person goes at least people are coming with them*, *which means three peoples wages are lost*. *So that is one thing*, *and then the distance*, *the decision to go*, *the expense*, *it is a huge*, *shall we say*, *loss of resources for them*.”

#### Geography and transport

In Aarohi, the scattered nature of the population over mountainous terrain was felt by most participants to be a core barrier affecting healthcare access. All participants felt that without the MMU, the distance required to travel by rural people to reach a PHC would be over 2 hours by vehicle on poor quality roads, with more specialised services such as ultrasound being significantly further. If the MMU was unavailable, several participants thought that most patients would wait until the next camp rather than travelling to hospital. One participant highlighted that this was particularly true of women, and that the MMU was often their only source of healthcare in this region.

Aarohi4: *“There is no ultrasound facilities here*, *only Almora*, *Almora is 35km*, *And Haldwani is nearly 85km*, *and Namita*. *The only here is Aarohi*, *there are not any facilities for ultrasound here*. *People are waiting for camp*.”

Aarohi1: *“Here we get a set of population that depends on us to take care of them*, *and the mobile medical unit heads into an area that is totally baron in terms of any medical help and being… since it also has investigations such as ultrasound and x-rays*. *It provides the people who live in really remote areas easy access to basic primary medical*.”

In SBMC, most participants felt that the physical distance of healthcare facilities from the rural population was a significant factor in preventing them accessing healthcare, with government hospitals being over 3 hours away by car. They felt that tribals were particularly affected by this due to high levels of poverty and poor access to transport. The lack of roads into tribal villages was highlighted by most participants, often limiting access to motorcycle or foot.

SBMC3: *“Yeah*, *transport now is government also*, *trying very hard to lay down the roads and all*, *but in villages we don’t have*, *road accessibility is also very less*. *And*, *transport system*, *all private transport system only*. *No public transport here*.”

Most SBMC participants highlighted that the main advantage of POCT would be bringing investigations closer to patients. Half of participants expressed that it was difficult to persuade patients to travel for tests due to non-direct costs, fear of direct costs, and potential distrust of outside medical communities. Half of participants felt that if they were able to diagnose patients with POCT on the spot, they would be more willing to engage with treatment or travel to receive treatment.

*SBMC5*: *“Solutions would be to have technology*, *which is available at the point of care*, *which would make it easier to make these diagnoses*. *For example*, *having a simple ultrasound*, *you can do a quick screening for gallstones*, *renal stones*, *kidney problems*, *or urinary obstruction*. *Or any lumps*, *or masses in the abdomen*, *or endoscopy that can be done in the field*.”

#### POCT maintenance and features

Whilst one Aarohi participant felt that available POCTs were serviced and maintained adequately, most participants felt that the devices often broke suddenly and were too complex to fix on site. They expressed that repair teams would have to travel from Delhi and would not always be available to come immediately, meaning that when POCT breaks down it is unpredictable how long it will be inoperative for.

Aarohi5: *“Basically*, *all the equipment needs servicing*. *And we*, *and we live in the rural areas*, *if any problems come during mobilisation to another place*, *we have to contact the service engineer person*, *and sometimes they come and sometimes they cannot arrive in the right time*.”

One cause for this frequent breaking of the POCT equipment was put down to the quality of the roads in the region by most participants. They felt the constant moving and vibrations caused by travel along often small and bumpy roads caused damage to the delicate machinery.

*Aarohi3*: *“Yeah*, *the equipment*, *is number 1 is that it is mobile bases*, *so it travels at least 400 to 500km a month*, *so the equipment*, *we keep facing problems with the equipment*.”

All SBMC participants highlighted the importance of POCT being portable due to poor quality roads in many rural villages. A few participants suggested that the devices must be simple to be able to use in field locations and stressed that POCT would ideally be battery operated due to a lack of electricity sources.

#### Gender and sex determination

Most Aarohi participants felt that sex selection–the practice of foetal abortion based on gender preference–remained an active issue. One participant suspected that some practitioners were still practicing sex selection within the state, but most felt that legislation had positively impacted on this practice here. Gender featured less heavily in the SBMC discussions compared to Aarohi. Many participants felt that sex selection was still an issue in India in general, however one participant felt that in tribals it was not an issue, and often sex ratios were reversed.

SBMC3: *“Yes*, *usually*, *among tribals there is no discrimination*. *Tribals even*, *male*, *female ratio is almost equal*, *and is some areas*, *it is more than the male child*, *girl child is*. *It is the major problem among non-tribal populations*.”

#### Human resources and specialist workforce

Most Aarohi participants felt that human resources were a significant limiting factor in their ability to fully utilise POCUS and provide adequate healthcare. One participant highlighted that organising specialist staff could be complex and expensive. Many participants highlighted radiologists as a significantly limited resource, and that without specific radiologists present, they were unable to use POCUS due to complex regulations.

Aarohi6: *“Other than the ultrasound machine*, *like suppose the ultrasound machine breaks down*, *or the radiologist comes from very far*, *because these villages are in remote areas*, *so the radiologist has to come a long way*. *So*, *for some reason if he cannot come*, *so*, *then we are helpless*.”

Most SBMC participants expressed that a key barrier for patients accessing testing was lack of availability. Half of participants highlighted that there were no large government hospitals within the region, and a few participants felt that the smaller PMCs were understaffed and have a propensity to shut down frequently, leaving service gaps. One participant believed that there may not be a radiologist working within the entire region.

SBMC5: *“…I mean in the sense there*, *they* (PHCs) *don’t cater to all the rural population because only the people around that centre can really utilise the services*. *So*, *what happens is there are various kinds of health providers*, *private practitioners*, *native medicine practitioners*, *even what you call as quacks*, *who then fill in the gaps*.”

#### Government regulation

In both locations, the sub-theme of government regulation was raised as a limiting factor for the use of POCUS by many participants. They highlighted that the regulations were strict, cumbersome, and complex. In Aarohi, two participants highlighted that due to the regulations, only specifically trained radiologists could use POCUS, only two POCUS devices could be registered to one radiologist, and only five named radiologists could be registered to any single POCUS device. Most participants felt that regulation was required to prevent sex selection; however, a few participants felt that the complexity of the regulations created difficulties for small rural organisations, often limiting their ability to use POCUS for these vulnerable group of people.

Aarohi3: *“Oh an issue*, *of course of course*, *it* (regulation) *has been an issue for us*, *like in multiple ways*. *It is not just about*, *because the law is very much complex*, *and it does not allow the doctor to*, *say practice in more than two places*, *two facilities*, *in just two facilities they can do this*. *And even then*, *also*, *you must be doing this silent record*, *and other things are attached with this*. *And particularly the problem is*, *the ultrasound is complex*, *they made the law complex because of the complex problem that is existing around it*. *The complex problem is the sex determination*.”

In SBMC, several participants felt that due to the disparities in human and physical medical resources experienced between urban and rural locations, the regulations unintentionally discriminated against rural, scattered populations by limiting their access to ultrasound in comparison to urban populations. A few participants believed that regulations needed to change to empower rural populations, such as by expanding those who are allowed to use POCUS devices.

SBMC5: *“Yes*, *in the sense that in the urban area there would be radiologists who would do non-antenatal or non-obstetric ultrasounds*. *So*, *it is easy to access*, *while in the rural area*, *it is only mostly*, *only obstetricians*, *who have ultrasound*. *And they are not allowed to do*, *I mean they are highly regulated*. *So*, *there is no question of them doing non-obstetric ultrasounds*, *and so the patients who need non-obstetric ultrasounds then get lost in that process*.”

Several participants highlighted the increased documentation pressures created by the regulations, and one participant had concerns over the effect the law had on migrant workers, as formal ID was required for POCUS, which often excluded this population.

Aarohi6: *“…see government has made it necessary to have proper ID*. *Only then can the radiologist be allowed to do the ultrasound*. *So*, *sometimes*, *when it were*, *patients do not have proper ID*, *like the migrant labourers*, *the Nepalese labourers… So*, *they are denied ultrasound because they do not have ID*. *And*, *they are pregnant*, *or they have some gynae problem*, *so… especially if they are young*, *female*, *or childbearing age*. *So*, *ultrasound is not done*.”

#### Telemedicine

In Aarohi, two participants felt that telemedicine would be a useful tool to overcome the lack of radiologists on site to interpret POCUS, but both were sceptical this could be managed under current regulations. One participant indicated that they had considered telemedicine to provide an on-call doctor, but they had felt that the mobile network in the region was not of high enough quality to proceed.

In SBMC, most participants believed telemedicine might be a useful way of supporting the medical team, as specialists are not always readily available in person within rural locations. However, a few participants were also concerned that the mobile network would be unable to facilitate this service.

SBMC3: *“Yes*, *telemedicine there is a role*. *But the main problem for tele-medicine is the connectivity in rural areas*.”

## Discussion

In this assessment of need for increased access to POCUS and the barriers to increasing access for POCUS within hard-to-reach communities in rural India, our study highlights that POCUS can be utilised effectively in these settings, but there are multiple intrinsic and extrinsic factors that need to be optimised to grasp the technology’s full potential. The difficulties of gaining research data on ‘hard-to-reach’ populations and the limitations that are often associated with this are well documented, yet the benefit of having data that reflects these needs is invaluable for policy makers [29]. Given these difficulties, there are some limitations on the interpretation of the results shown here. The qualitative data represents the articulated views of participants involved with two medical teams supplying healthcare to rural populations in Uttarakhand and Jharkhand via mobile camps. Therefore, extrapolation of these results to other circumstances should be done with care. Notably, both camps were operated by NGOs–although Aarohi did have a formal relationship with the local government–meaning that these results may not necessarily correlate with government-run facilities in similar areas. These sampling techniques are not representative, so this data cannot give information on how commonly these views would be shared by other similar organisations providing similar services. No data was collected directly from patients or non-service users from the community, and therefore information given concerning them represents the articulated opinions of the participants about them alone and may differ from the lived experience of the local community. The quantitative data collected from Aarohi represents patients who presented to the camp, so may have missed patients who presented to other locations and may have been less likely to collect data on emergency presentations that needed immediate treatment. Notably, the quantitative data collected from SBMC was incomplete, as data was only recorded when it was felt it may be useful directly in the future for the management of the patient or for other reasons, with other patients who attended the clinic not being directly recorded. Therefore, the quantitative data from SBMC should be cautiously interpreted.

With these considerations in mind, these results suggest that POCUS is an acceptable investigation amongst rural, hard-to-reach populations, which was frequently used for abdominal presentations when available. Several location-specific barriers were highlighted through the study. Participants highlighted that health seeking behaviour was reduced in rural regions compared to equivalent urban populations; in particular, the tribal populations of Jharkhand were felt to face significant barriers to health seeking. Further research would be required to demonstrate how this difference in culture and structure might impact the introduction of POCUS. Another barrier identified included the increased distance from healthcare in this setting, with limited transport and infrastructure options available to overcome this, which were particular barriers to healthcare in both settings. Most participants with access to POCUS found that the ability to bring investigations to the patient was a significant advantage. To overcome the remote, hard-to-reach nature of these environments, POCUS is likely to need to be highly portable, robust to transport damage, easily repairable, and battery operated. The results show that where POCUS was available, there appeared to be an increase in coding of confirmed, rather than suspected, lithiasis-related disease and complications during pregnancy. Due to a lack of follow-up or true comparison control data, it is not possible to draw conclusions on if this is due to population differences, if these are accurate diagnoses, or if these diagnoses led to improved clinical management.

These results also suggest that sex determination and gender are still key issues within rural, hard-to-reach Indian communities. They further indicate that the PNDT Act is indeed having the unintentional effect of inequitably limiting access of rural communities to diagnostic ultrasound, in comparison to their urban counterparts. This seems to be in part due to the limited human resources with permission to operate POCUS in these regions, mixed with reduced incentives for those willing to travel due to the limitations it places on their ability to provide ultrasound services in their own region.

The National Rural Healthcare Mission of India launched in 2005, detailing the national governments commitment to reducing inequity and creating a patient-centred, decentralised rural healthcare system for improved access for rural people [30]. In 2015, the Indian government launched the “Free Diagnostics Service Initiative”, stating its commitment towards providing essential diagnostics free of charge [31]. Building on this and the existing essential medicines list, the Indian Council of Medical Research launched a National Essential Diagnostics List of essential diagnostics that should be made available at each public health infrastructure level down to individual villages through a mix of POCT devices and ‘hub and spoke’ diagnostic services [32]. Interestingly ultrasound technology is not recommended until secondary care level and above. These results suggest that POCUS at the primary care level would fit well with the current aims of the government if barriers around regulation can be safely overcome.

Telemedicine was highlighted as a way of overcoming limitations of constrained human resources within rural settings; however, both locations highlighted that limited telecommunications infrastructure was a perceived barrier to its introduction more broadly, and that PNDT regulations would severely limit its usefulness for POCUS. Previous studies have shown the capacity for digital image transfer to allow rapid and improved ultrasound image interpretation in multiple developing countries [12–14,33]. Further work in this area, in combination with Indian authorities, might be useful to see if this technology could be combined within the current laws to improve access to diagnostic ultrasound.

### Policy recommendations and conclusions

#### Regulatory barriers

Awareness should be raised of the concerns held by rural practitioners that current regulations around ultrasound are unintentionally, but inequitably, reducing access to POCUS for rural populations in comparison to urban populations in India. Finding ways of overcoming these barriers should be of high priority and will need to be explored in conjunction with Indian regional and national authorities if it is to succeed. This could include addressing the limited availability of POCUS at PHCs and Community Healthcare Centres.

#### Disease burden

Surgical abdominal conditions continue to be a significant burden within rural Indian communities. Urological conditions were felt by participants to be increased in their rural regions, and the reasons for this may benefit from further research.

#### Clinical investigations in hard-to-reach populations

POCT is an ideal way to reduce barriers and expand access to healthcare in hard-to-reach, rural locations with difficult geographical features and limited transport options.

#### Advantageous POCT design features

POCT and POCUS should be highly portable, robust, easily repairable, and battery operated.

#### Telemedicine

For telemedicine to be fully utilised, signal quality will have to be proven to be reliable within these regions as currently there is little confidence in its abilities.

## References

[1] Meara J, Leather A, Hagander L, Alkire B, Alonso N, Ameh E, et al. Global Surgery 2030: Evidence and solutions for achieving health, welfare, and economic development. The Lancet. Lancet Publishing Group; 2015. pp. 569–624. doi: 10.1016/S0140-6736(15)60160-X 25924834

[2] Dare A, Ng-Kamstra J, Patra J, Fu S, Rodriguez P, Hsiao M, et al. Deaths from acute abdominal conditions and geographical access to surgical care in India: a nationally representative spatial analysis. Lancet Glob Health. 2015;3: e646–e653. doi: 10.1016/S2214-109X(15)00079-0 26278186

[3] The Royal College of Surgeons of England. Emergency General Surgery—Commissioning Guide. 2014.

[4] Mazzei MA, Guerrini S, Squitieri NC, Cagini L, Macarini L, Coppolino F, et al. The role of US examination in the management of acute abdomen. Crit Ultrasound J. 2013;5. doi: 10.1186/2036-7902-5-S1-S6 23902801 PMC3711740

[5] Obajimi MO, Atalabi MO, Ogbole GI, Adeniji-Sofoluwe AT, Agunloye AM, Adekanmi AJ, et al. Abdominal ultrasonography in HIV/AIDS patients in southwestern Nigeria. BMC Med Imaging. 2008;8. doi: 10.1186/1471-2342-8-5 18312644 PMC2275264

[6] Raman S, Somasekar K, Winter RK, Lewis MH. Are we overusing ultrasound in non-traumatic acute abdominal pain? Postgrad Med J. 2004;80. doi: 10.1136/pgmj.2003.013805 15016943 PMC1742958

[7] Maru D, Schwarz R, Andrews J, Basu S, Sharma A, Moore C. Turning a blind eye: the mobilization of radiology services in resource-poor regions. Glob Health 2010 61. 2010;6: 1–8. doi: 10.1186/1744-8603-6-18 20946643 PMC2964530

[8] Maksimovic R, Berumen A. Innovative Technology in Addressing Global Health Issues: the WHO Perspective. 2011.

[9] World Health Organization. Training in diagnostic ultrasound: essentials, principles and standards: report of a WHO study group. World Health Organization; 1998. p. 46 p. 9659004

[10] Bélard S, Tamarozzi F, Bustinduy AL, Wallrauch C, Grobusch MP, Kuhn W, et al. Review article: Point-of-care ultrasound assessment of tropical infectious diseases-a review of applications and perspectives. American Journal of Tropical Medicine and Hygiene. 2016. doi: 10.4269/ajtmh.15-0421 PMC4710450 26416111

[11] Edvardsson K, Ntaganira J, Åhman A, Sengoma JPS, Small R, Mogren I. Physicians’ experiences and views on the role of obstetric ultrasound in rural and urban Rwanda: a qualitative study. Trop Med Int Health. 2016;21. doi: 10.1111/tmi.12718 27125579

[12] Shokoohi H, Raymond A, Fleming K, Scott J, Kerry V, Haile-Mariam T, et al. Assessment of Point-of-Care Ultrasound Training for Clinical Educators in Malawi, Tanzania and Uganda. Ultrasound Med Biol. 2019;45: 1351–1357. doi: 10.1016/j.ultrasmedbio.2019.01.019 30904246

[13] Spencer J, Adler R. Utility of Portable Ultrasound in a Community in Ghana. J Ultrasound Med. 2008;27: 1735–1743. doi: 10.7863/jum.2008.27.12.1735 19022999

[14] Epstein D, Petersiel N, Klein E, Marcusohn E, Aviran E, Harel R, et al. Pocket-size point-of-care ultrasound in rural Uganda—A unique opportunity “to see”, where no imaging facilities are available. Travel Med Infect Dis. 2018;23: 87–93. doi: 10.1016/j.tmaid.2018.01.001 29317333

[15] Wydo S, Seamon M, Melanson S, Thomas P, Bahner D, Stawicki S. Portable ultrasound in disaster triage: a focused review. Eur J Trauma Emerg Surg. 2016;42: 151–159. doi: 10.1007/s00068-015-0498-8 26038019

[16] Jha P, Kumar R, Vasa P, Dhingra N, Thiruchelvam D, Moineddin R. Low female[corrected]-to-male [corrected] sex ratio of children born in India: national survey of 1.1 million households. Lancet Lond Engl. 2006;367: 211–218. doi: 10.1016/S0140-6736(06)67930-0 16427489

[17] Phutke G, Laux T, Jain P, Jain Y. Ultrasound in rural India: a failure of the best intentions. Indian J Med Ethics. 2019;4: 39–45. doi: 10.20529/IJME.2018.041 29976548

[18] Subramanian S, Selvaraj S. Social analysis of sex imbalance in India: before and after the implementation of the Pre-Natal Diagnostic Techniques (PNDT) Act. J Epidemiol Community Health. 2009;63: 245–252. doi: 10.1136/jech.2008.078477 19033295

[19] Christianson A, Zimmern R, Kristoffersson U, Schmidtke J, Kent A, Raouf R, et al. Health needs assessment for medical genetic services for congenital disorders in middle- and low-income nations. J Community Genet. 2013;4: 297–308. doi: 10.1007/s12687-013-0150-4 23794314 PMC3739852

[20] Hooper J, Longworth P. Health needs assessment workbook. 1st ed. Health Development Agency (UK); 2002.

[21] Tong A, Sainsbury P, Craig J. Consolidated criteria for reporting qualitative research (COREQ): a 32-item checklist for interviews and focus groups. Int J Qual Health Care. 2007;19: 349–357. doi: 10.1093/intqhc/mzm042 17872937

[22] World Bank Group. India States Briefs–Jharkhand. 2016.

[23] World Bank Group. India States Briefs–Uttarakhand. 2017.

[24] Ministry of Health & Family Welfare Government of India. National Health Profile 2018. 2018.

[25] Ritchie J, Spencer L. Qualitative data analysis for applied policy research. 1st editio. In: Bryman A, Burgess B, editors. Analyzing Qualitative Data. 1st editio. London: Routledge; 1994.

[26] Green J, Thorogood N. Qualitative methods for health research. 3rd editio. Los Angeles: SAGE; 2014.

[27] Parkinson S, Eatough V, Holmes J, Stapley E, Midgley N. Framework analysis: a worked example of a study exploring young people’s experiences of depression. 2015;13: 109–129. doi: 10.1080/14780887.2015.1119228

[28] Swallow V, Newton J, Van Lottum C. How to manage and display qualitative data using “Framework” and Microsoft Excel. J Clin Nurs. 2003;12: 610–612. doi: 10.1046/j.1365-2702.2003.00728.x 12790875

[29] Bonevski B, Randell M, Paul C, Chapman K, Twyman L, Bryant J, et al. Reaching the hard-to-reach: A systematic review of strategies for improving health and medical research with socially disadvantaged groups. BMC Med Res Methodol. 2014;14. doi: 10.1186/1471-2288-14-42 24669751 PMC3974746

[30] Chokshi M, Patil B, Khanna R, Neogi S, Sharma J, Paul V, et al. Health systems in India. J Perinatol Off J Calif Perinat Assoc. 2016;36: S9–S12. doi: 10.1038/JP.2016.184 27924110 PMC5144115

[31] Ministry of health and family welfare. National Health Mission—Free Diagnostics Service Initiative. New Dehli; 2015.

[32] Indian Council of Medical Research. National Essential Diagnostics List. New Dehli; 2019.

[33] Swanson J, Plotner D, Franklin H, Swanson D, Bolamba V, Lokangaka A, et al. Web-Based Quality Assurance Process Drives Improvements in Obstetric Ultrasound in 5 Low- and Middle-Income Countries. Glob Health Sci Pract. 2016;4: 675. doi: 10.9745/GHSP-D-16-00156 28031304 PMC5199182

